# Characterization and Sensing of Inert Gases with a High-Resolution SPR Sensor

**DOI:** 10.3390/s20113295

**Published:** 2020-06-10

**Authors:** Zhenchao Liu, Jinlong He, Sailing He

**Affiliations:** 1 Centre for Optical and Electromagnetic Research, National Engineering Research Center for Optical Instruments, Zhejiang University, Hangzhou 310058, China; lzc_archer@zju.edu.cn; 2 Ningbo Research Institute, Zhejiang University, Ningbo 315100, China; 3 College of Modern Science and Technology, China Jiliang University, Hangzhou 310018, China; jlhe@cjlu.edu.cn

**Keywords:** inert gas, SPR, sensing, refractive index, high-resolution

## Abstract

It is generally difficult to characterize inert gases through chemical reactions due to their inert chemical properties. The phase interference-sensing system based on high-resolution surface plasmon resonance (SPR) has an excellent refractive index detection limit. Based on this, this paper presents a simple and workable method for the characterization and detection of inert gases. The phase of light for the present SPR sensor is more sensitive to the change in the external dielectric environment than an amplitude SPR sensor. The limit of detection (LOD) is usually in the order of 10^−6^ to 10^−7^ RIU, which is superior to LSPR (Localized Surface Plasmon Resonance) sensors and traditional SPR sensors. The sensor parameters are simulated and optimized. Our simulation shows that a 36 nm-thick gold film is more suitable for the SPR sensing of inert gases. By periodically switching between the two inert gases, helium and argon, the resolution of the system is tested. The SPR sensing system can achieve distinguishable difference signals, enabling a clear distinction and characterization of helium and argon. The doping of argon in helium has a detection limit of 1098 ppm.

## 1. Introduction

Inert gases are stable and thus often difficult to react with other substances. Because of the inactive chemical nature of inert gas, it is commonly used as a protective gas in industrial production and scientific research. In general, it is hard to characterize them by direct chemical reactions. Common indirect detection methods include emission spectroscopy [1], mass spectrometry [2], and gas chromatography [3,4]. These methods require some tedious and time-consuming steps. The testing equipment is usually large and expensive, and has limited sensitivity. For example, emission spectroscopy requires the electrical or thermal excitation of inert gases. This process will increase the cost of detection; mass spectrometry detection instruments are bulky, have high detection costs, and require vacuum during detection, so the instrument needs to be evacuated to a vacuum state in advance. The gas chromatography detection step is complicated and time-consuming and also requires a bulky chromatographic analysis instrument. Thus, it is urgent to find a simple, fast, and low-cost characterization method.

Plasmon sensors are rapidly becoming a key instrument in the high-sensitivity analysis of samples [5,6,7,8]. Plasmon sensors have become a promising detection technology for chemical and biological analytes due to them being high-sensitivity, label-free, and supportive of in-situ measurements. In general, the limit of detection (LOD) of the phase surface plasmon resonance (SPR) sensor is one to two orders of size lower than other plasmon sensors. Thus, it is more widely used in the field of high-sensitivity analysis [9,10,11,12,13]. Wood [14] first discovered the SPR phenomenon when studying metallic diffraction gratings. Before this, the Localized Surface Plasmon Resonance (LSPR) phenomenon of metal nanoparticles has been used to change the color of glass but the mechanism has not been explained. It was not until Fano [15] gave a theoretical explanation of the SPR phenomenon that SPR began to become a research hotspot. The first active excitation and utilization of the SPR phenomenon started from the two structures that stimulated the SPR phenomenon proposed by Kretschmann [16] and Otto [17]. Between them, the Kretschmann structure has been used these days because of its simple structure, ease of control, low price, and good stability. Since then, the SPR has begun to emerge in the field of sensing and analysis. Liedberg [18] first proposed the use of SPR sensors for gas sensing. After that, SPR sensors were quickly applied to the field of liquid-phase biosensing. SPR sensors are also widely used in researching gas sensing. Berrier has proposed a NO_2_ gas sensor based on the SPR effect. By depositing a layer of NO_2_ gas-sensitive layer composed of 5,10,15,20-Tetrakis(4-hydroxyphenyl)-21H,23H-porphine (2H-OHTPP) on the sensing surface, it can be used to sense NO_2_ [19]. At the same time, the enrichment of nanoporous silicon has been demonstrated to enhance the sensitivity of NO_2_ gas sensing [19]. A layer of ultra-thin ZnO (15 nm) on the surface of the SPR sensor has been proved to be sensitive to NH_3_ and NO_2_ gases under different light conditions, and can be used in the dual sensing of NH_3_ and NO_2_ [20]. There are also related studies of gas sensing based on the LSPR effect of metal particles. For example, by controlling the deposition time of RF-PECVD to control the shape of Ag nanoclusters, researchers can tune the intensity of the LSPR resonance peaks of the Ag nanoclusters and enable the highly sensitive sensing of CO in the air [21]. A planar nanocomposite film that uses a composite of silver nanoparticles and a titanium dioxide film (Ag-TiO_2_) has been proposed to sense ozone gas [22]. The research on SPR gas sensing is not limited to traditional metal materials. Some researchers use the SPR effect of other materials for gas sensing. Graphene plasmon has been used to enhance the absorption spectrum, which can be applied to sense the gas molecules SO_2_, NO_2_, N_2_O, and NO [23]. There are also related studies on gas sensing based on optical fiber SPR sensors. A graphene-based long-period fiber grating (LPFG) SPR sensor has been demonstrated suitable for methane gas sensing [24]. The LPFG sensor has a sensing surface covered with graphene, which enhances the plasmon resonance intensity, and can realize a sensitive detection of methane. Most of the above gas sensing based on the SPR sensor requires a pre-chemical modification of the sensing surface, but surface chemical modification has complicated operation steps and difficult regeneration. It also cannot be directly used for sensing and characterizing inert gas. Gas sensing based on the graphene plasma effect can avoid chemical modification, but the gas still needs to have a characteristic absorption peak, and the inert gas is difficult to distinguish and characterize from the perspective of a characteristic absorption peak due to its stable chemical properties. Some researchers have proposed a direct inert gas detection method based on LSPR spectral sensors [25]. However, the sensitivity of LSPR is low and the LOD is usually 10^−5^ RIU [26,27], which cannot meet the requirements of the high-sensitivity detection and characterization of inert gases. At the same time, LSPR-based sensors need some bulky spectrum analysis instruments and take longer to scan a spectrum.

Therefore, we proposed a simple and highly sensitive new method for characterizing and sensing inert gases. The usual chemical modification methods are incapable of the characterization and sensing of inert gas, the detection cost by means of instruments such as mass spectrometers is too high, and gas chromatography is complicated and time-consuming. We proposed a phase-interference-SPR sensor that can characterize and sense the inert gas at high sensitivity from the angle of the refractive index. In the experiment, we used two inert gases, helium and argon, as examples. When periodically changing the inert gases, we obtained different signal responses far above the noise level, enabling a highly sensitive distinction and characterization of different types of inert gas. At the same time, through simulation we optimized the gold film thickness of the SPR sensor to improve the sensitivity of the device. In addition to this, we have also conducted experiments on the ability of the system to resolve the doping of trace inert gases for a couple of two inert gases mixed in traces. The detection limit can reach up to 1098 ppm. We believe that this article will provide a new idea and method for the characterization and sensing of inert gases in a simple, fast, and highly sensitive manner.

## 2. Material and Methods

### 2.1. Optical Configuration of SPR Sensor

**Figure 1:**
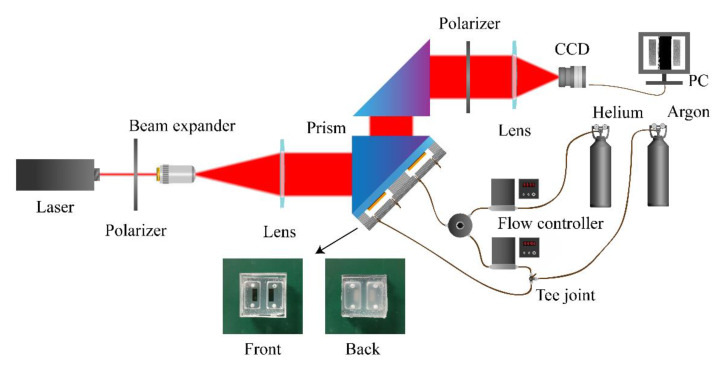
Phase interference surface plasmon resonance (SPR) sensor for inert gas sensing.

The configuration of the SPR system for gas sensing is shown in Figure 1. The system uses a semiconductor laser to provide a light source with 632.8 nm wavelength, a polarizer (Union Optic, Wuhan, China, SHP1050) to provide a linear polarization light, and a combination of a 20× objective lens (OLYMPUS, Tokyo, Japan, MPLN20x) and a focusing lens (Union Optic, Wuhan, China, 4001030032) to provide an extended beam light source. A prism (Union Optic, Wuhan, China, RAP0025) coupling structure provides an SPR excitation of Kretschmann type. An analyzer (Union Optic, Wuhan, China, SHP1050) is used to provide polarized interference between TM-polarized light and TE-polarized light. Finally, an imaging lens is combined with a CCD (Thorlab, Newton, MA, USA, DCU224M) for imaging sensing.

### 2.2. Configuration of Inert Gas Sensor Chip

As shown in Figure 1, we made a special sensing chip for inert gases and coupled it with the prism surface using an index-matching liquid (OLYMPUS, Tokyo, Japan, F30CC). The dimension of the gas sensor chip is 25.4 × 25.4 × 15.0 mm, and the size of the gas chamber is 15.0 × 8.0 × 3.0 mm. The chip has three main components. The first layer is a gold film with N-BK7 glass substrate. The second layer is an air chamber layer. The air chamber layer is composed of two perforated PMMA substrates. The first layer of perforated PMMA substrate is bonded with the gold film substrate by UV glue, and the perforation is a sensing space for inert gases. Two perforated PMMA substrates are sealed together with UV glue to provide a closed air chamber space. As shown in the actual chip diagram in Figure 1, the third layer is a fixed layer of the gas duct, which is composed of a relatively soft PDMS polymer (PDMS: curing agent = 10:1, heat-cured for 1 h at 85 °C), and is bonded with the second PMMA substrate by industrial glue. The soft texture and small openings provide the function of fixing gas conduits and preventing gas leakage. To seal PDMS and PMMA tightly, the contact surfaces need to be flat. Thus, we use ultra-flat silicon wafers as the substrate to make molds when curing PDMS.

### 2.3. Gas Cylinder Installation and Gas Path Configuration

We use a flow meter (SEVENSTAR, Beijing, China, D07-19C) to control the flow rate of helium and argon (Hangzhou Metalworking Materials Co., Ltd., Hangzhou, China) to achieve different content ratios. Among them, the helium is divided into two paths through a three-way joint, one of which flows into the flow meter to mix with the argon, while the other directly flows into the channel of the reference gas chamber.

## 3. Results and Discussion

### 3.1. Sensor Parameter Optimization

**Figure 2:**
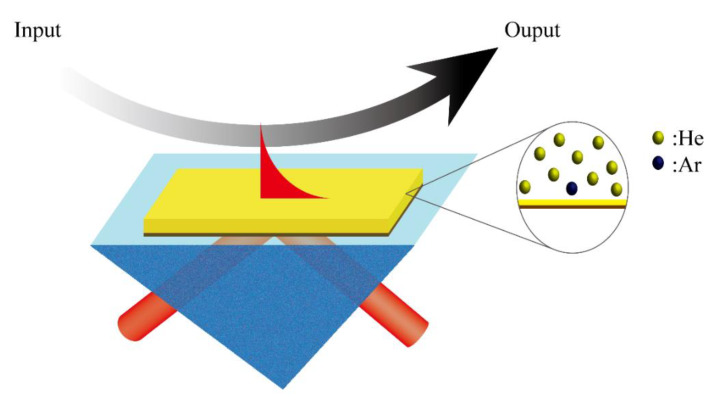
Schematic diagram of the proposed method for detecting inert gas: Kretschmann prism-coupled SPR sensor; enlarged view of a part showing a mixture of helium and argon.

**Figure 3:**
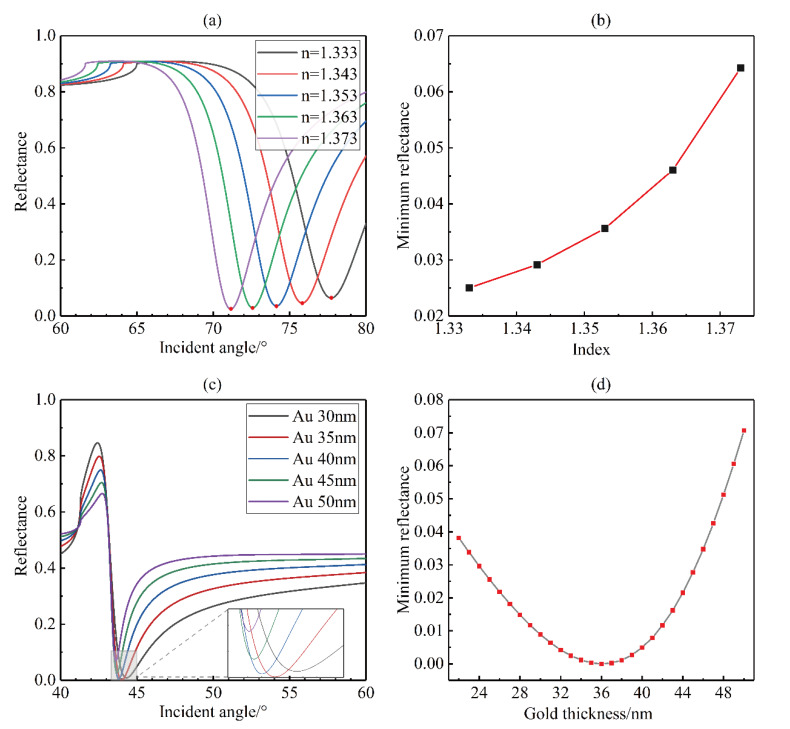
Simulation of optimized parameters for gas sensing: (**a**) intensity reflectance curves as the incident angle changes for different dielectric environments; (**b**) the minimum values of the intensity reflectance as the refractive index changes; (**c**) intensity reflectance curves as the incident angle changes for different thicknesses of gold film (light gray area at the bottom is enlarged to the small block diagram); (**d**) the minimum values of the intensity reflectance as the gold film thickness varies.

There are inherent refractive index differences between inert gas molecules. SPR sensors are very sensitive to the effective refractive index on the sensing surface. When the type of inert gas changes (for example, from helium to argon) or is lightly doped (as shown in Figure 2), the effective refractive index on the sensing surface changes. Small changes of the effective refractive index on the sensing surface will cause a change in the reflectance response of the SPR sensor. In general, the optimal thickness of the gold film for liquid-phase SPR sensing is around 50 nm. We performed a numerical simulation based on the multilayer film transmission matrix at 50 nm. As shown in Figure 3a, the resonance angle of the reflected light decreases as the effective refractive index on the gold surface increases. At the same time, the lowest point of the reflectance curve will also move upward, as shown in Figure 3b. This indicates that less energy is coupled with the surface plasmon resonance mode, and consequently causes a decrease in the sensitivity of our SPR sensor. There are different optimal sensing thicknesses for different refractive index detection ranges. Figure 3c shows the simulated SPR reflectance curve as a function of the incident angle for different thicknesses of the gold film. Here, we use the refractive index of helium at 20 °C (*n* = 1.000035). The dotted box is an enlarged view of the lowest points of intensity reflectance. Compared with liquid phase detection, when the detection sample is replaced with a gas, the thickness of the gold film corresponding to the optimal sensitivity of the sensor is changed because the refractive index becomes smaller. The thinner gold film thickness is more suitable for gas sensing. When the gold film thickness is around 35 nm, the sensitivity is the best, as the resonance reflectance is minimal. Considering the limitations of the actual process, we used 1 nm as the thickness interval of the gold film in the simulation. Finally, as shown in Figure 3d, it can be found that a 36 nm gold film thickness has a better sensitivity and is more suitable for the detection of inert gases. The adhesion of the gold film to the glass substrate is relatively poor. We sputtered a layer of 5 nm Cr onto the glass substrate as the adhesion layer of the gold film. In this section, through simulation, we demonstrate that due to the change in the sensing medium type, the SPR resonance peak will move and, at the same time, it will also cause a loss of sensitivity. Finally, we obtained the optimal sensor film thickness for inert gas sensing, which is different from the thickness parameters commonly used in previous studies.

### 3.2. Distinguish between the Response of Inert Gas (Helium and Argon)

The difference in the refractive indices among different inert gases is the basis for sensing. During the experiment, we need to adjust the system to the SPR excitation angle of helium in advance. The phase change of the SPR sensor is much more severe than that of the light intensity, so it is necessary to adjust the SPR excitation angle precisely. Here, we constructed a double prism system, as shown in Figure 1. The coupling structure of Krestchmann prism was placed at the center of the turn table. Then, a corresponding prism with the opposite orientation was placed. During the small adjustment of the SPR angle, it is no longer necessary to adjust the imaging equipment. Thus, we adjust the incident angle to a position where the gray value of the image is the smallest. This position is the optimal SPR excitation angle. When argon fills the measurement channel, the refractive index changes. This causes the phase of the TM mode in the reflected light to change, and thereby the interference light intensity changes. 

**Figure 4:**
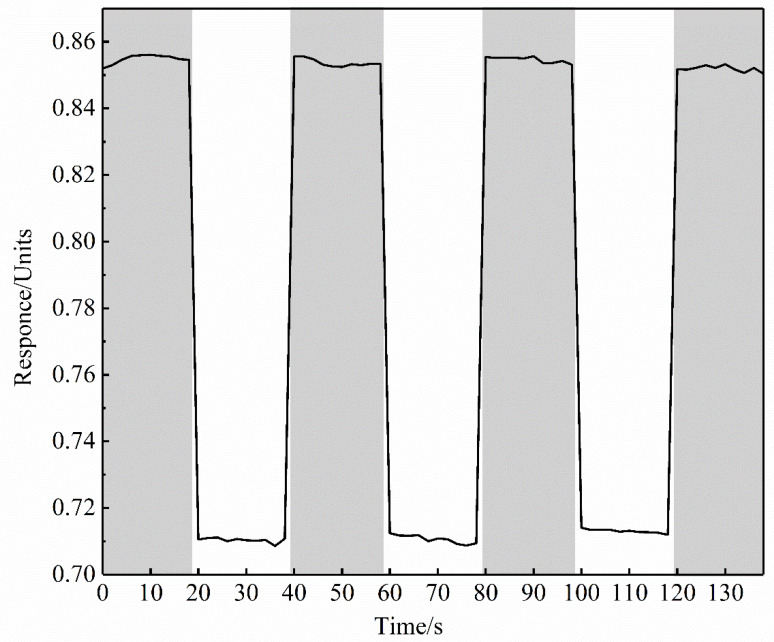
On-off response of helium and argon. The light gray area is argon and the white area is helium.

By periodically changing the inert gas in the measurement channel, we obtained the on-off curve shown in Figure 4, where the dark area is argon and the white background area is helium. Here, we used a simple method to eliminate the fluctuation of the light source and reduce the noise. We take the ratio of the interference light intensity of the measurement channel to that of the reference channel as the final signal response. The average responses of helium and argon were 0.710 and 0.856, respectively, with a difference of 0.146. Considering that the sensing noise in the helium is approximately 6.7 × 10^−4^, we achieved a clear characterization and differentiation. The spatial distribution of the light intensity is non-uniform, and this will cause the grayscale value (on the CCD) of the reference and measurement channels to be unequal. In the present work, we added a glycerin liquid sealing device to the air outlet duct to avoid gas leakage and water vapor interference. The glycerin seal also guarantees a relatively constant pressure environment for the system. Unfortunately, the switching response of the same gas shown in Figure 4 is slightly shifted. This may be due to a slight difference in gas pressure after gas switching, resulting in a slightly different refractive index. In this section, we periodically change the inert gas in the measurement channel to obtain different sensing responses, whose response value is much greater than the noise, enough to distinguish and characterize different types of inert gas. It is reasonable to extend this to all inert gases, which can be differentiated and characterized based on the sensing system and method provided in this article.

### 3.3. Resolving Power of Two Inert Gas Doping

**Figure 5:**
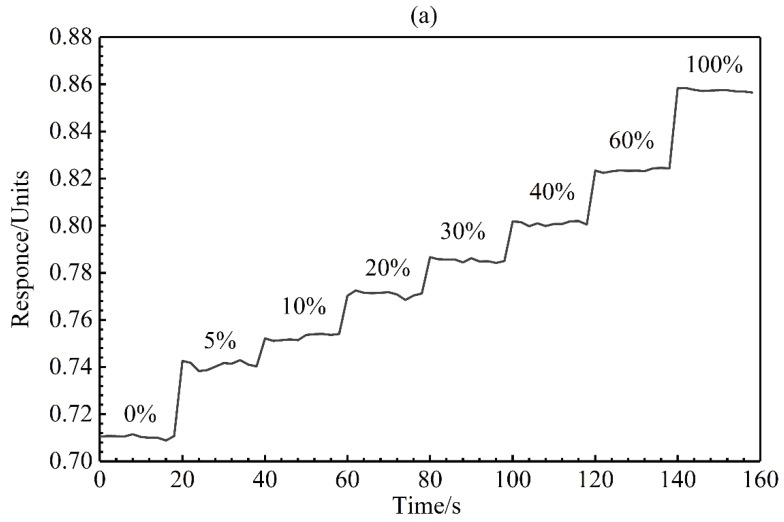

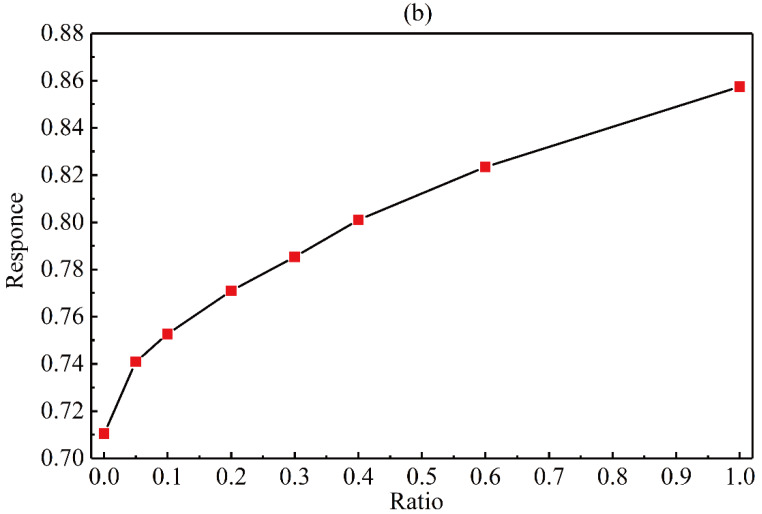
SPR response of helium and argon in different ratios: (**a**) 0% means full of helium, 100% means full of argon. (**b**) Response curve as the ratio varies.

When it is necessary to detect the doping of one of the two inert gases based on the sensing system mentioned in this article, we have verified its resolving power. The present SPR system will get different response values for helium and argon, and thus two different inert gases can be distinguished. We further studied the high-resolution capabilities of the system. When only helium and argon are present in the test sample, the ability of the system to resolve a small amount of argon in the background of helium was further demonstrated. In this part, we ensure that the interferer has only one inert gas, helium, and detect the minute content of another inert gas, argon. Thus, we used a flow mass spectrometer to control the flow rate of the two inert gases and mix them in the mixing chamber. Different ratios of flow rate will produce different proportions of the mixed gas. Then, we passed the mixture into the measurement channel for detection. Helium is still selected as the reference. As shown in Figure 5a, the mixing ratio ranges from 5% argon doped in helium to full argon. Ten tests were performed to determine the SPR response curve with each gas concentration. The gradient curve shows that as the mixing ratio increases, the system response gradually increases. The calibration curve shown in Figure 5b is the relationship between the average response and the mixing ratio. The standard error for Figure 5b is presented in the Supplementary Materials (Figure S1). The non-linearity of the system response may be due to the increase in the mixing ratio, which causes the phase change to become saturated. And a set of gray images with different gas mixture ratio is presented (Figure S2). Due to the limited control accuracy of the flow meter, the smallest mixing ratio we can adjust is 5%. Considering the response noise in the helium background as the sensing noise of the system (approximately 6.7 × 10^−4^), the limit of detection (LOD) can be extrapolated. The sensor response change for 5% argon doping is 0.0305 and the system noise is approximately 6.7 × 10^−4^. If we consider the system noise as the resolution limit the estimated LOD is approximately 1098 ppm (5% × 6.7 × 10^−4^/0.0305). The difference between the inherent refractive index of helium and argon is 2 × 10^−4^ RIU. Therefore, it is estimated that the refractive index difference of a mixed gas with a small concentration of 5% argon relative to helium is 1 × 10^−5^ RIU (2 × 10^−4^ RIU × 5%). Similarly, the refractive index difference of 1098 ppm doping is 2.2 × 10^−7^ RIU. According to the additive nature of the refractive index, a mixing ratio of 1098 ppm corresponds to a refractive index difference of 2.2 × 10^−7^ RIU. Thus, the LOD of the SPR sensing system after thickness parameter optimization in this paper is 2.2 × 10^−7^ RIU.

### 3.4. Repeatability, Reproducibility, and Response Time

As shown in Figure 4, by repeatedly switching between helium and argon, we found that the response values of helium and argon are basically the same during repeated measurement. The repeatability of the system is very high as the fluctuation error of the periodically changing response value is much smaller than the response value. At the same time, it also ensures that the sensor chip has a good reproducibility and can be used repeatedly. Since the system detects optical variables without any chemical modification, its response time depends mainly on the detection speed of the instrument. Considering the exposure time, the response time of the CCD is in the order of ms. The system CCD reads the data 10 times and takes the average value as a set of measurement data. Therefore, the response time of the system is about 10 ms.

## 4. Conclusions

Based on phase-interference-SPR sensors of high sensitivity, we have proposed a simple and workable method to characterize and detect inert gases to avoid complex and expensive detection methods such as mass spectrometry and gas chromatography. The detection time is about 20 seconds. The sensing principle is small refractive index differences between different inert gases. To detect inert gas, the thickness of the gold film is optimized. Through simulation, we found that 36 nm gold film gives the best sensitivity.

By periodically changing the type of inert gas, the switching response curves of helium and argon have been demonstrated experimentally. The response change is much larger than the noise of the sensing system. The system can achieve a clear distinction and characterization. The system’s ability to resolve small amounts of argon in the background of helium has also been demonstrated. We have used a flow mass spectrometer to control the flow rate of the two inert gases and mixed them to produce mixed gases of different proportions. The detection result of the gradient change of argon content has shown that the detection limit of argon doping in helium can reach 1098 ppm. Compared with previous studies using the LSPR spectrum to characterize and distinguish inert gas [23], the present SPR phase interference sensors have higher sensitivity and are able to better distinguish inert gas, even when one kind of inert gas is mixed in a very small amount into another kind of inert gas. Therefore, it is suitable for the detection of inert gas, which requires a higher sensitivity. The gold thickness is optimized to achieve the higher-sensitivity characterization and detection of inert gases. It also provides a possible method for analyzing the content of inert gas with high sensitivity. An extremely low detection limit of 1098 ppm has been achieved. The future research direction can enhance the selectivity by means of the size of the inert gas molecules.

## Supplementary Materials

The following are available online at https://www.mdpi.com/1424-8220/20/11/3295/s1: Figure S1: The presentation of the standard error; Figure S2: A set of gray images with different gas mixture ratio.
