# Supplementary material for: Characterization and Sensing of Inert Gases with a High-Resolution SPR Sensor
Source: Sensors (Basel). 2020 Jun 10;20(11):3295. doi: 10.3390/s20113295 (PMC7309052; doi:10.3390/s20113295)
Supplement: Supplementary file 1 [file sensors-20-03295-s001.pdf]

1. The numerical calculation.

The simulation software is MATLAB, and we simulated based on the multilayer film transmission matrix. The following are the related equations.

Transfer matrix M is defined as:

$$M = \prod_{k=1}^{N} M_k = \begin{pmatrix} M_{11} & M_{12} \\ M_{21} & M_{22} \end{pmatrix}$$

and:

$$M_k = \begin{pmatrix} \cos\beta_k & (-i\sin\beta_k)/q_k \\ -iq_k\sin\beta_k & \cos\beta_k \end{pmatrix}$$

$$q_k = \frac{\left(\varepsilon_k - n_1^2\sin^2\theta_1\right)^{1/2}}{\varepsilon_k}$$

$$\beta_k = \frac{2\pi}{\lambda} d_k \left(\varepsilon_k - n_1^2\sin^2\theta_1\right)^{1/2}$$

where ε_k and d_k mean the dielectric constant and thickness of the k-th layer, respectively.

And the reflection coefficient R_p can be expressed as:

$$R_p = \frac{\left(M_{11} + M_{12}q_N\right)q_1 - \left(M_{21} + M_{22}q_N\right)}{\left(M_{11} + M_{12}q_N\right)q_1 + \left(M_{21} + M_{22}q_N\right)}$$

2. The standard error for Figure 5b.

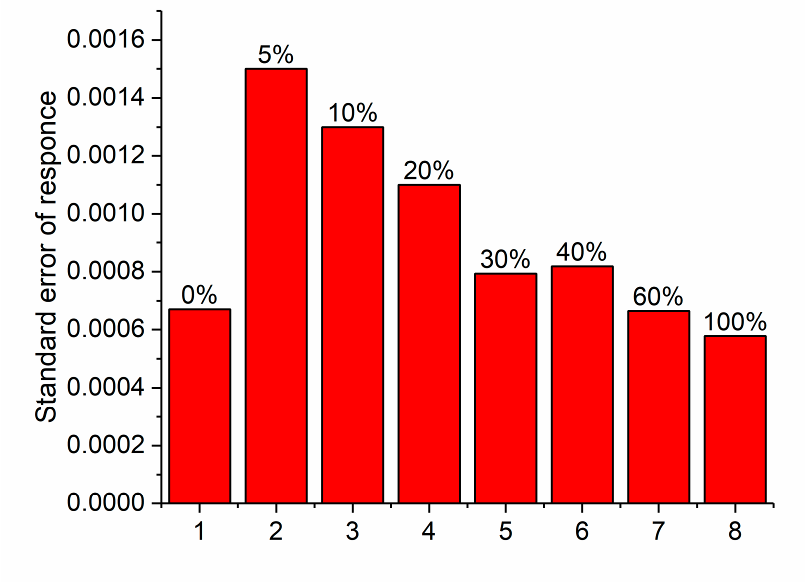

Figure S1. The standard error for Figure 5b.

3. A set of gray images with different gas mixture ratio.

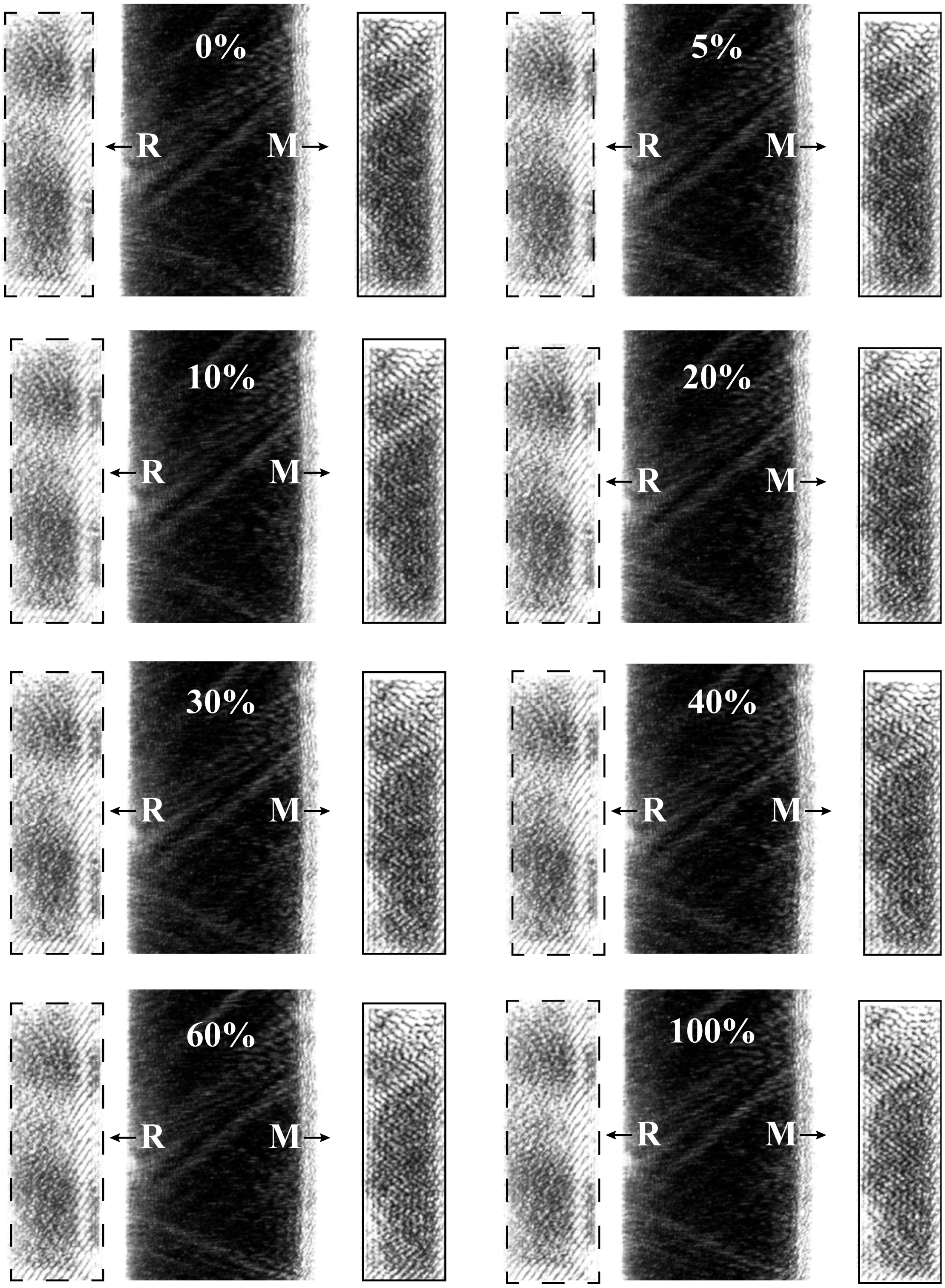

Figure S2. A set of gray images with different gas mixture ratio, where R represents the reference channel, and M represents the measurement channel.
